# Supplementary material for: Corticosterone Impairs Hippocampal Neurogenesis and Behaviors through p21-Mediated ROS Accumulation
Source: Biomolecules. 2024 Feb 23;14(3):268. doi: 10.3390/biom14030268 (PMC10968297; doi:10.3390/biom14030268)
Supplement: Supplementary file 1 [file biomolecules-14-00268-s001.zip › biomolecules-2845197-supplementary.pdf]

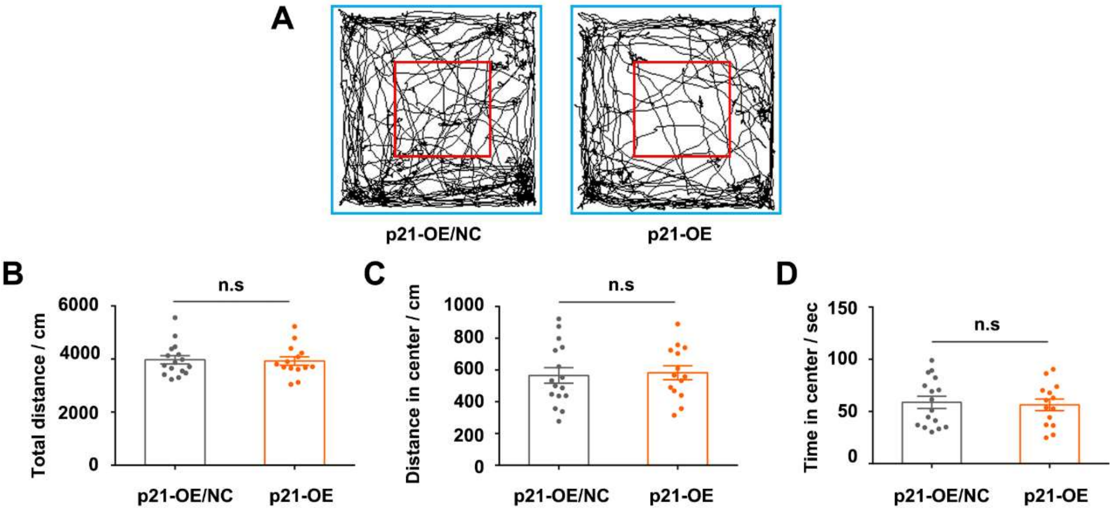

Supplementary Figure 1. P21-overexpression does not influence the locomotor activity of mice. A. Representative trajectory diagram of the mice in the open field test. **B.** Total distance traveled by mice in the whole area of the apparatus. **C, D.** Distance traveled **(C)** and time spent **(D)** by mice in the central zone of the open filed.

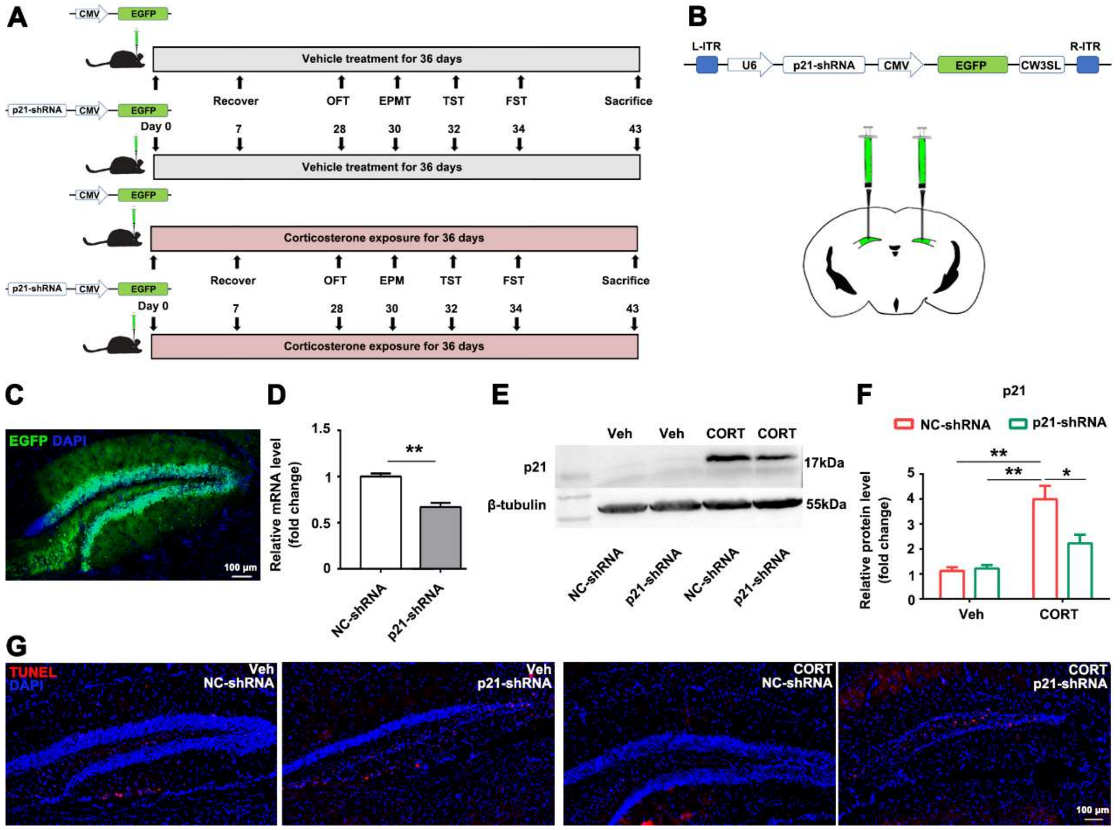

Supplementary Figure 2. P21-knockdown caused apoptosis of granular neurons. A. Study design. **B.** Schematic representation of stereotaxic injection of AAV vectors into the bilateral DG areas of mice brain. **C.** Visualization of AAV vectors in hippocampus by mNeonGreen fluorescence. **D.** Analysis of p21 mRNA expression level. **E, F.** Western blot analysis **(E)** and quantification **(F)** of p21 protein expression level. **G.** Representative images of TUNEL staining within the dentate gyrus (DG) area. Data are presented as mean ± SEM, n = 3 per group. The Student's t test or one-way ANOVA was performed to compare the means for two or four groups, respectively. *$P < 0.05$, **$P < 0.01$. NC: negative control.

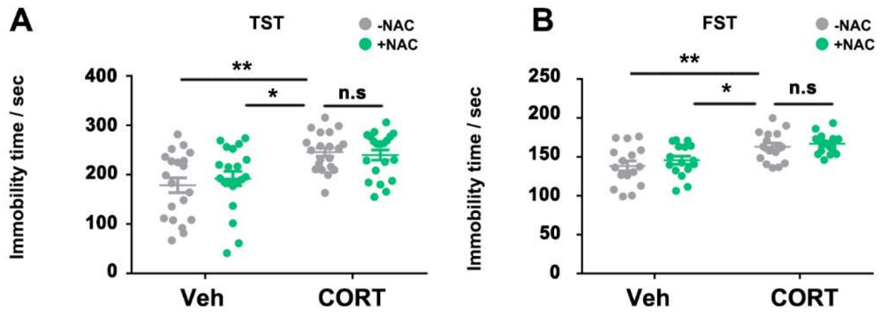

Supplementary Figure 3. Antioxidant does not attenuate CORT-induced depressive-like behaviors. Immobility time of the mice in the tail suspension test **(A)** and the forced swimming test **(B)**. Data are presented as mean ± SEM, n = 22 per group for behavior tests. Statistical comparisons were performed by two-way ANOVA. *P < 0.05, **P < 0.01, ***P < 0.001, n.s.: no significance.

Supplementary Table 1. The list of DEGs analyzed by PPI network. (Related to Figure.2E)

Gene name	Gene set name	CORT3	CORT2	CORT1	WT3	WT2	WT1
Ddit4	intrinsic apoptotic signaling pathway by p53 class mediator	142.58	146.02	146.77	78.21	71.16	90.92
Ifi204	intrinsic apoptotic signaling pathway by p54 class mediator	0.25	0.25	0.12	0	0.03	0
Pmaip1	intrinsic apoptotic signaling pathway by p55 class mediator	1.68	2.09	1.86	0.6	0.65	0.65
Cdkn1a	intrinsic apoptotic signaling pathway by p56 class mediator	49.82	63.35	70.7	10.97	9.76	14.21
Rrp8	intrinsic apoptotic signaling pathway by p57 class mediator	10.92	10.27	14.76	6.84	5.41	6.05
Parp1	oxidative phosphorylation	92.79	88.06	83.66	69.45	64.88	65.67
Ncf1	oxidative phosphorylation	8.06	8.06	6.93	4.99	4.16	5.82
Setx	oxidative phosphorylation	10.64	9.92	9.48	9.05	7.62	7.06
Ucp2	oxidative phosphorylation	77.47	73.13	68.58	30.55	28.01	35.4
Apoe	oxidative phosphorylation	4747.87	4863.09	4470.74	5659.6	5347.6	5769.65
Cyp26b1	oxidative phosphorylation	7.06	7.04	5.04	7.96	8.26	7.5
Trp53inp1	oxidative phosphorylation	5.07	4.44	5.63	2.58	1.97	1.92
Duox2	oxidative phosphorylation	0.12	0.36	0.19	0.07	0.01	0.11
Xdh	oxidative phosphorylation	9.39	9.24	8.28	3.37	3.97	3.8
Akt2	oxidative phosphorylation	55.18	57.93	47.23	67.01	60.93	66.92
Pdk4	oxidative phosphorylation	7.3	7.46	8.47	4.09	3.51	3.64
Gab1	oxidative phosphorylation	8.07	7.98	7.15	13.87	8.99	10.15
Emilin2	positive regulation of inflammatory response	7.03	6.34	6.77	4.79	2.12	2.73
Mt2	positive regulation of inflammatory response	1446.99	1546.45	1304.05	978.18	880.05	946
Nppc	positive regulation of inflammatory response	12.65	18.29	15.53	6.96	5.83	7.91
Pla2g3	positive regulation of inflammatory response	6.16	6.45	8.31	0.87	1	0.81
Peli1	positive regulation of inflammatory response	21.29	20.57	20.52	15.43	15.54	14.29
Serpina3n	positive regulation of inflammatory response	88.12	89.39	76.25	62.1	53.47	53.99
Cd59a	positive regulation of inflammatory response	7.74	5.37	8.03	2.73	3.59	3.3
Il1r1	positive regulation of inflammatory response	4.72	4.53	4.16	3.26	3.18	3.35
Il7r	positive regulation of inflammatory response	0.35	0.23	0.38	0.07	0.04	0.12

Gene name	Gene set name	CORT3	CORT2	CORT1	WT3	WT2	WT1
Tgm2	positive regulation of inflammatory response	16.17	17.63	13.37	6.19	5.83	11.37
Ctsh	positive regulation of inflammatory response	23.25	13.58	18.33	12.72	8.92	15.66
Serpina3f	positive regulation of inflammatory response	0.87	1.28	0.31	0.04	0	0.11
Lrg1	positive regulation of inflammatory response	10.47	9.45	5.47	1.99	0.66	4.91
Irak2	positive regulation of inflammatory response	17.52	22.82	18.11	12.34	13.29	14.58
Spns2	positive regulation of inflammatory response	78.47	82.96	80.05	59.81	57.15	60.47
Ldlr	positive regulation of inflammatory response	11.45	12.37	13.14	9.34	8.24	9.78
Stat5a	positive regulation of inflammatory response	2.08	2.84	1.88	0.71	0.55	1.37
Arg2	positive regulation of inflammatory response	5.34	4.45	5.94	3.04	3.5	3.09
Fcgr3	positive regulation of inflammatory response	27.16	24.5	19.64	14.55	14.11	20.98
Havcr2	positive regulation of inflammatory response	1.83	1.73	2.41	0.99	1.59	1.11
Il20rb	positive regulation of inflammatory response	5.2	4.4	4.22	3.75	2.72	2.87
Adamts1	positive regulation of inflammatory response	10.81	9.4	9.22	6.35	5.27	6.12
Fkbp5	positive regulation of inflammatory response	114.7	126.81	122.95	48.86	44.37	45.49
Abca1	positive regulation of inflammatory response	5.97	6.94	6.34	5.09	3.79	4.3
Shb	positive regulation of inflammatory response	15.25	12.57	18.39	9.93	11.26	11.85
Ada	positive regulation of inflammatory response	4.71	4.83	4.76	0.93	0.73	0.61
Cebpa	positive regulation of inflammatory response	16.5	15.52	15.62	10.78	12.25	11.75
Nfkbia	positive regulation of inflammatory response	69.91	72.2	75.46	30.23	22.36	30.96
Kdr	regulation of phosphatidylinositol 3 kinase signaling	2.05	2.18	1.67	4	3.26	3.23

Gene name	Gene set name	CORT3	CORT2	CORT1	WT3	WT2	WT1
Slc9a3r2	regulation of phosphatidylinositol 4 kinase signaling	35.32	39.27	37.24	45.26	42.6	46.63
Fn1	regulation of phosphatidylinositol 5 kinase signaling	16.8	19.25	16.72	19.26	21.66	24.92
Pdgfrb	regulation of phosphatidylinositol 6 kinase signaling	6.12	5.88	6.5	7.96	6.89	7.4
Vegfa	regulation of phosphatidylinositol 7 kinase signaling	24.76	30.07	30.86	36.92	48.35	39.71
Flt1	regulation of phosphatidylinositol 8 kinase signaling	13.37	11.81	11.29	15.39	13.71	14.38
Slc9a3r2	regulation of phosphatidylinositol 9 kinase signaling	35.32	39.27	37.24	45.26	42.6	46.43
Trem2	regulation of phosphatidylinositol 10 kinase signaling	25.46	26.11	20.39	31.6	35.66	34.74
Sox9	regulation of phosphatidylinositol 11 kinase signaling	7.56	7.17	7.92	16.04	12.07	12.3
Tek	regulation of phosphatidylinositol 12 kinase signaling	2.79	2.92	2.45	4.41	3.95	3.74
Adora3	regulation of phosphatidylinositol 13 kinase signaling	0.3	0.42	0.13	0.62	0.72	0.29
Pdgfb	regulation of phosphatidylinositol 14 kinase signaling	19.47	17.05	17.15	22.29	22.97	22.84
Ddr1	regulation of phosphatidylinositol 15 kinase signaling	40.24	45.97	40.03	56.47	41.88	52.64
Reln	regulation of phosphatidylinositol 16 kinase signaling	34.4	27.68	29.44	36.58	31.86	43.01
Ascl1	response to virus	4.14	3.99	5.92	5.66	5.86	7.22
Tagap	response to virus	1.29	1.07	1.66	0.39	0.3	0.42
Nt5c3	response to virus	39.63	43.62	51.45	27.76	27.35	26.08

Gene name	Gene set name	CORT3	CORT2	CORT1	WT3	WT2	WT1
Nmb	response to virus	5.41	11.9	5.98	4.4	3.07	4.99
Nlrp6	response to virus	0.75	1.45	1.09	0.78	0.4	0.34
Tlr7	response to virus	1.7	1.69	1.78	0.55	0.52	0.71
Acod1	response to virus	0.12	0.06	0.24	0	0.03	0
Bcl2l1	response to virus	127.4	131.6	132.18	96.68	89.43	97.39
Sox18	fibroblast proliferation	10.25	11.18	10.17	16.61	16.24	17.45
Pax6	fibroblast proliferation	3.83	4.93	4.73	6.59	5.43	5.84
Gjc2	fibroblast proliferation	12.68	16.86	12.76	21.07	14.92	19.65
Prrx1	fibroblast proliferation	0.77	0.67	1.05	2.11	1.36	1.17
Vegfd	fibroblast proliferation	3.27	3.23	2.82	1.9	2.02	1.53
Fgfr2	fibroblast proliferation	15.99	12.8	13.17	27.11	22.9	22.07
Gli3	fibroblast proliferation	0.87	1.04	1.04	1.51	1.33	1.51
Serpine1	fibroblast proliferation	1.45	1.9	1.7	0.4	0.46	0.38
Gli1	fibroblast proliferation	0.73	0.26	0.87	0.81	1.47	1.36
Ptch1	fibroblast proliferation	7.33	6.78	6.73	10.56	8.34	8.88
Nr2e1	fibroblast proliferation	5.68	4.85	7.55	7.91	7.61	7.06
Zfp36l1	fibroblast proliferation	9.73	9.4	10.61	14.89	12.92	12.33
Fzd9	fibroblast proliferation	2.81	3.37	2.46	5.38	4.62	4.07
Sox2	fibroblast proliferation	18.74	21	19.03	34.44	29.64	29.25
Rarg	fibroblast proliferation	3.63	3.33	4.05	5.58	4.67	5.96
Sox10	fibroblast proliferation	16.59	18.21	18.69	40.75	29.76	41.4
Pim1	fibroblast proliferation	1.7	1.38	2.22	2.49	2.53	2.63
Cx3cr1	fibroblast proliferation	8.84	8.99	7.02	20.99	20.32	21.4
Hdac5	fibroblast proliferation	115.32	126.21	114	154.06	141.21	143.59
Sall1	fibroblast proliferation	4.05	4.27	4.77	8.07	5.64	6.24
Tgfb1	fibroblast proliferation	4.23	3.72	3.21	6.87	5.44	6.87
Mc4r	adaptive thermogenesis	7.76	8.52	7.59	4.39	3.4	2.97
Gadd45g	adaptive thermogenesis	46.87	43	43.65	25.63	28.73	30.95
Npr3	adaptive thermogenesis	7.75	7.78	7.06	5.24	3.95	4.14
Dio2	adaptive thermogenesis	13.72	15.05	15.46	12.62	10.68	10.4
Il4ra	adaptive thermogenesis	4.6	3.54	2.09	1.86	1.85	2.65
Ebf2	adaptive thermogenesis	0.59	0.47	0.12	0.22	0.06	0.07
Prlr	adaptive thermogenesis	2.36	1.23	0.85	0.77	0.43	0.71
Cebpb	adaptive thermogenesis	21.69	21.06	21.56	14.22	15.66	17.14
Lpin1	adaptive thermogenesis	11.67	12.05	12.76	9.19	8.44	8.65
Zbtb7b	adaptive thermogenesis	20.3	21.58	21.19	15.32	14.73	17.83
Fermt1	regulation of WNT signaling pathway	1.07	0.23	0.7	0.64	0.52	2.89
Arntl	regulation of WNT signaling pathway	12.44	14.27	13.19	16.25	15.51	17.23
Lmbr1l	regulation of WNT signaling pathway	3.75	6.06	6.15	11.92	8.08	7.76

Gene name	Gene set name	CORT3	CORT2	CORT1	WT3	WT2	WT1
Grem1	regulation of WNT signaling pathway	0.75	1.44	1.25	2.17	1.92	1.54
Tmem88b	regulation of WNT signaling pathway	24.23	29.51	24.24	38.23	25.88	29.42
Nkd1	regulation of WNT signaling pathway	7.1	9.39	10.59	10.81	9.26	12.09
Pin1	regulation of WNT signaling pathway	36.44	33.86	28.34	37.22	36.61	41.06
Prickle1	regulation of WNT signaling pathway	33.67	33.41	32.46	39.06	38.31	39.48
Egr1	regulation of WNT signaling pathway	82.55	77.15	87.9	106.77	99.8	110.21
Notum	regulation of WNT signaling pathway	0.49	2.36	0.95	2.29	1.5	2.34
Tle4	regulation of WNT signaling pathway	18.78	16.47	18.67	22.59	20.95	19.9
Rspo2	regulation of WNT signaling pathway	11.54	9.66	11.05	14.32	12.98	16.02
Sox4	regulation of WNT signaling pathway	5.05	5.67	6.41	7.88	6.48	7.78
Col1a1	regulation of WNT signaling pathway	1.02	0.73	0.74	1.22	1.26	2.33
Rnf43	regulation of WNT signaling pathway	0.88	0.93	1.36	1.66	1.33	1.57
Hhex	regulation of WNT signaling pathway	0.93	0.29	0.8	1.49	1.67	1.39
Sox13	regulation of WNT signaling pathway	3.64	3.95	3.73	5.39	5.39	5.56
Apcdd1	regulation of WNT signaling pathway	14.21	15.19	20.19	21.56	17.84	18.44
Sox7	regulation of WNT signaling pathway	0.48	0.52	0.47	1.22	0.67	1.15
Nrarp	regulation of WNT signaling pathway	11.27	12.4	15.14	17.11	15.27	14.14
Angptl4	response to starvation	5.4	5.56	6.28	1.62	1.42	1.95
Zfp36l1	response to starvation	9.73	9.4	10.61	14.89	12.92	12.33
Fas	response to starvation	3.48	2.15	2.4	1.55	0.8	1.15
Slc2a1	response to starvation	131.57	141.36	132.07	79.17	62.39	79.53

Supplementary Table 2. The list of primers used in this study.

qPCR primers		
Actb	forward	GCTATGTTGCTCTAGACTTCG
	reverse	GGATTCCATACCCAAGAAGG
Cdkn1a	forward	TTGCACTCTGGTGTCTGAG
	reverse	GTGATAGAAATCTGTCAGGCTG
Parp1	forward	GTGCAAACTACTGCCACAC
	reverse	AGTTCATACATGTTTCCAAGGG
Didt4	forward	CAGAGAAGAGGGCCTTGAC
	reverse	GTATGAGGAGTCTTCCTCCG
Trp53inp1	forward	TCACAGTCGGATATCAGCC
	reverse	ATCTTCTGGAGGAAGTAGTTTCC
Pmaip1	forward	CATAACTGTGGTTCTGGCG
	reverse	TCGTCCTTCAAGTCTGCTG
Akt2	forward	TCAGAAGTGGACACAAGGT
	reverse	AGGCTGTCATATCGGTCTG
